# Stunned by a Heatwave: Experimental Heatwaves Alter Juvenile Responsiveness to the Threat of Predation

**DOI:** 10.1002/ece3.71447

**Published:** 2025-05-14

**Authors:** Merel C. Breedveld, Oliviero Borgheresi, Alessandro Devigili, Clelia Gasparini

**Affiliations:** ^1^ Department of Biology University of Padova Padova Italy; ^2^ Department of Biology and Evolution of Marine Organisms Stazione Zoologica Anton Dohrn, Fano Marine Center Fano Italy; ^3^ National Biodiversity Future Center (NBFC) Palermo Italy

**Keywords:** climate change, heat stress, Poeciliidae, predation risk, prey behaviour

## Abstract

Heatwaves, increasingly prevalent in our rapidly changing climate, significantly impact animals with far‐reaching ecological and evolutionary consequences. One of the first responses in animals to stress, including heat stress, is behavioural change, and this can directly influence fitness and survival. Changes in anti‐predator behaviour are particularly critical, as they may compromise a prey's ability to evade predators, thus increasing predation risk and jeopardising survival. In the context of climate change, assessing anti‐predator reactions under ecologically relevant heat stress is thus crucial, especially during the vulnerable life stage of development. This study investigated the effects of a heatwave on anti‐predator responses in juvenile guppies ( *Poecilia reticulata* ). One‐month‐old guppies were subjected to a 5‐day experimental heatwave (32°C) or a control temperature (26°C). After the treatment, all individuals were tested at a common temperature (26°C) for anti‐predator behavioural responses and swimming performance, the latter serving as a proxy for physical condition. While heatwave exposure did not affect swimming performance, it significantly altered anti‐predator responses. Heatwave‐exposed juveniles exhibited a reduced freezing response and faster resumption of normal activity compared to control fish. Our findings demonstrate that heatwaves can modify prey's anti‐predator behaviours during critical developmental stages. This suggests that heatwaves may increase predation risk, potentially impacting survival rates and reshaping predator–prey interactions in the face of ongoing climate change.

## Graphical abstract

We assessed how a heatwave affects anti‐predator reactions of juvenile guppies ( *Poecilia reticulata* ), by tracking their activity in an open field setup. When presented with a predator cue, guppies that were previously exposed to a 5‐day experimental heatwave (32°C) displayed an attenuated freezing response and resumed normal swimming activity faster than guppies from a control temperature (26°C). The results show that heatwaves can change prey's anti‐predator behaviours, suggesting that heat stress induced by climate change may influence predation risk in the wild.

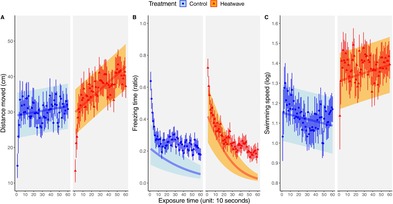

## 1 Introduction

Heatwaves, characterised as a period of abnormally hot weather (IPCC 2021), are occurring with an increasing frequency and intensity under ongoing climate change (Perkins et al. 2012; Ummenhofer and Meehl 2017; IPCC 2023). Beyond their direct impact on the survival of animals and humans (McKechnie and Wolf 2010; Xu et al. 2016; Piatt et al. 2020), heatwaves can induce subtle sublethal changes in physiology and behaviour (Buchholz et al. 2019; Stillman 2019). Behavioural changes are often the first response to altered environmental conditions (Wong and Candolin 2014) and can have important implications for reproduction and survival. Heatwaves have been shown to induce changes in a range of fitness‐related behaviours, including foraging behaviour (Funghi et al. 2019; Hemberger et al. 2023), mating behaviour (Breedveld et al. 2023; Grandela et al. 2024), parental care (Pilakouta et al. 2023; Barrett and Stein 2024) and anti‐predator behaviour (Cordonnier et al. 2023; Jawad et al. 2024). Sometimes, behavioural responses may buffer organisms against the effects of climate change and heatwaves (Wong and Candolin 2014; Stillman 2019), such as increased foraging or drinking effort to meet increased energetic or hydric demands during heat stress (Naga Raja Kumari and Narendra Nath 2018; Osborne et al. 2020; Dezetter et al. 2022). Likewise, parents may increase parental care to protect their offspring from temperature variation (Grew et al. 2019). On the other hand, behavioural plasticity may not be enough to offset the fitness costs of heatwaves (Le Roux et al. 2024), and some behavioural responses may instead have negative consequences, jeopardising health or survival in the long term. If individuals shift their diet in response to heat in a way that compromises nutritional balance (Carreira et al. 2016), or if they start ignoring predation cues (Gutiérrez et al. 2023), these changes could negatively impact populations.

Among the behavioural traits that could be impacted by heatwaves, anti‐predator behaviours deserve more consideration. Beyond the direct impact of anti‐predator behaviours on organisms' survival chances (Alcock 2009), climate induced alterations in anti‐predator behaviours may have implications on ecological (predator–prey) interactions, with potential cascading effects on communities and ecosystems (Laws 2017; Rivest et al. 2019; Rahman and Candolin 2022). Thus far, some studies indicate that heatwaves can reduce the behavioural responsiveness of prey to predators or predator cues, thereby potentially increasing predation risk. Snails reduce predator avoidance behaviour, through reduced climbing height, during heatwaves (Jawad et al. 2024); great tits respond less to conspecific mobbing calls, an anti‐predator signal, during hot days (Cordonnier et al. 2023); damselfish show a decreased escape response, allowing predators to approach closer before fleeing, and escape distance under elevated temperature (Allan et al. 2015); and shorebirds show a lowered flight initiation distance, thereby accepting a higher predation risk, during periods of high environmental temperature (Gutiérrez et al. 2023). Some other studies find that heatwaves do not alter the behavioural responsiveness of prey to predators (Smolinský and Gvoždík 2014; Sentis et al. 2017; Stahlschmidt et al. 2024). Aphids, for example, typically occupy the higher, more nutrient‐rich parts of their microhabitat (i.e., the apex of a plant) but move to lower plant parts in response to predator presence. This microhabitat shift, however, remains unaffected by heat shocks (Sentis et al. 2017). Yet other studies point to heatwave‐induced shifts in other behavioural traits of prey organisms (e.g., foraging behaviour, behavioural thermoregulation; Du Plessis et al. 2012; Funghi et al. 2019; Leu et al. 2021), or in predator behaviour (Evans et al. 2020; Fromant et al. 2021), all of which can change predator–prey interactions and thus alter predation risk. The effects of heatwaves on prey's anti‐predator responses may be especially pronounced when heatwaves are experienced during ontogeny, since immature individuals are more susceptible and vulnerable to predation and, at the same time, they are still learning how to recognise and react to predators (Paradis et al. 1996; Kelley and Magurran 2003b; Lingle et al. 2008; Hoy et al. 2015). Under current climate change, which includes an increase in extreme temperature events, such effects of heatwaves on anti‐predator responses could increase predation rates and thereby impact survival rates in prey populations, with potential cascading effects on communities. Yet, research into the effects of heatwaves on anti‐predator behaviour is thus far scarce.

Here, we investigated the effects of an experimental heatwave during development on anti‐predator responses in juveniles, using the guppy, *Poecilia reticulata* , as a model species. Guppies are small freshwater fish native to Trinidad and Tobago but are now found around the globe (Deacon et al. 2011). Guppies inhabit small streams and are preyed upon by several predators, including birds and other fish (Kelley and Magurran 2003a; Templeton and Shriner 2004). It is an ideal species to study the impact of heatwaves on anti‐predator responses for several reasons. First, this species has been extensively used in behavioural ecology, and in particular in studies on anti‐predator behaviour, with well‐established protocols for eliciting and measuring anti‐predator responses in the lab and in the field (e.g., Seghers 1974; Kelley and Magurran 2003a; Evans et al. 2007; Swaney et al. 2015). Observed behavioural responses to experimental predator cues include hiding, freezing, reducing activity, and increased shoaling behaviour (Kelley and Magurran 2003a; Fischer et al. 2015; Kimbell and Morrell 2015; Swaney et al. 2015), which are typical anti‐predator behaviours seen in the wild (Magurran and Seghers 1994; Magurran 2005). Second, heatwave occurrence is an ecologically relevant problem for this species—as for many others—in its natural habitat (Stephenson et al. 2014; Angeles‐Malaspina et al. 2018). Third, as ectotherms, guppies are generally sensitive to changes in temperature (Angilletta 2009), making their anti‐predator behaviour likely to be affected by heatwaves, in line with our recent findings showing heatwave‐induced alterations in guppy sexual behaviour (Breedveld et al. 2023).

We tested whether a heatwave affects anti‐predator responses in 1‐month‐old juvenile *P. reticulata* using a split clutch approach, where siblings were randomly assigned to either the treatment (heatwave) or the control. Following the heatwave (or control) exposure, we tested each individual's anti‐predator behaviour at a common temperature (26°C) in an open field set up, in response to a combination of a visual and an olfactory predator cue. A combination of different cues (i.e., visual and olfactory) provokes a stronger response in guppies (Brown et al. 2004; Evans et al. 2007), and allows us to distinguish between immediate but short reactions typically triggered by visual stimuli (e.g., Evans et al. 2004), and longer lasting responses driven by chemical stimuli (Nordell 1998; Brown et al. 2004). In addition to the anti‐predator response, we measured swimming endurance, which is associated with body condition, using a flow chamber (e.g., Breedveld et al. 2023), and performed a ‘capture test’ (e.g., Evans et al. 2007) to estimate each fish's physical ability to evade an approaching predator, i.e., its manoeuvrability. Based on our previous findings in this species (Breedveld et al. 2023) and studies in different species (Cordonnier et al. 2023; Jawad et al. 2024), we predicted that swimming performance would not differ between the two groups, but that guppies exposed to the heatwave would exhibit a reduced behavioural responsiveness to predator cues compared to controls.

## 2 Materials and Methods

### 2.1 Overview of the Experimental Design

Juvenile *P. reticulata* (35 ± 3 days old) were exposed to either an experimental heatwave (32°C) or a control temperature (26°C) for 5 days, and their behaviour was assessed the following day. The temperatures were chosen based on our previous study on sublethal effects of heatwaves in adult guppies (Breedveld et al. 2023), and they align with the heatwave definition used by Sales et al. (2018), which describes a heatwave as the temperature exceeding the local average by 5°C–7°C for at least 5 days. The heatwave temperature is the same as that used in a previous study on this species (Breedveld et al. 2023), is within the range of naturally occurring temperatures for the species (Reeve et al. 2014), and is well below the CTmax for the species (Grinder et al. 2020).

Newborn guppies were raised in family groups of four siblings until they were 1‐month old. Each family group was then split into two, randomly assigning two juveniles to the control and the other two to the heatwave treatment. One day after the end of the treatment, the anti‐predator response was measured at a common temperature (26°C) in an open field arena using both a visual and a chemical predator cue (hereafter referred to as short‐ and long‐term anti‐predator response, respectively). Following this, predator evasion capacity was assessed using a capture test. Finally, swimming endurance was assessed in a flow chamber. Body size (standard length, SL in mm, through image analysis of digital photography) and weight (g) of each fish were also measured to use these traits as covariates in our statistical models when appropriate. In addition, body size was used alongside body weight to quantify body condition, using Fulton's body condition index (Kotrschal et al. 2011).

### 2.2 Fish Origin and Maintenance

All fish used in this experiment were descendants of wild‐caught guppies from the Tacarigua River in Trinidad. This site is a high‐predation locality where guppies coexist with a diverse array of predators (Magurran and Seghers 1994). Fish are maintained at standard conditions for the species (see Breedveld et al. 2023), with water temperature maintained at 26°C ± 1°C and illumination provided with a 12 L: 12 D cycle. All fish were fed twice a day with a mix of brine shrimp ( *Artemia salina* nauplii) and commercial dry food (Duplarin). Visibly pregnant females were moved to individual tanks and monitored closely until birth, allowing for the collection of newborns used for the experiment.

### 2.3 Heatwave Treatment

A total of 104 newborn guppies, from 20 different families, were raised under equal density of 4 fish under standard laboratory conditions (as described above) for 1 month. When they were 35 days old (±3 days), two juveniles from each family were assigned to the heatwave treatment (32°C for 5 days) while the other two juveniles were assigned to the control treatment (26°C for 5 days). Our split clutch approach is important to minimise confounding effects, as anti‐predator responses in this species have been shown to have a heritable component (O'Steen et al. 2002).

On the morning of day 1 of the treatment (at 10:00), the fish were placed into perforated containers (1.5 L) which were immersed in a larger, 15 L treatment tank. The containers were partially perforated to allow water exchange between the container and the surrounding water in the treatment tank. The water was constantly maintained at either the heatwave temperature (32°C) or at the control temperature (26°C), using aquarium heaters (300 W NEWA Therm VTX). On the afternoon of day 5 (at 16:00), the containers with the fish were removed from the treatment tank and left at room temperature (i.e., 26°C) until the following morning.

### 2.4 Behavioural Assays

#### 2.4.1 Anti‐Predator Response

**FIGURE 1:**
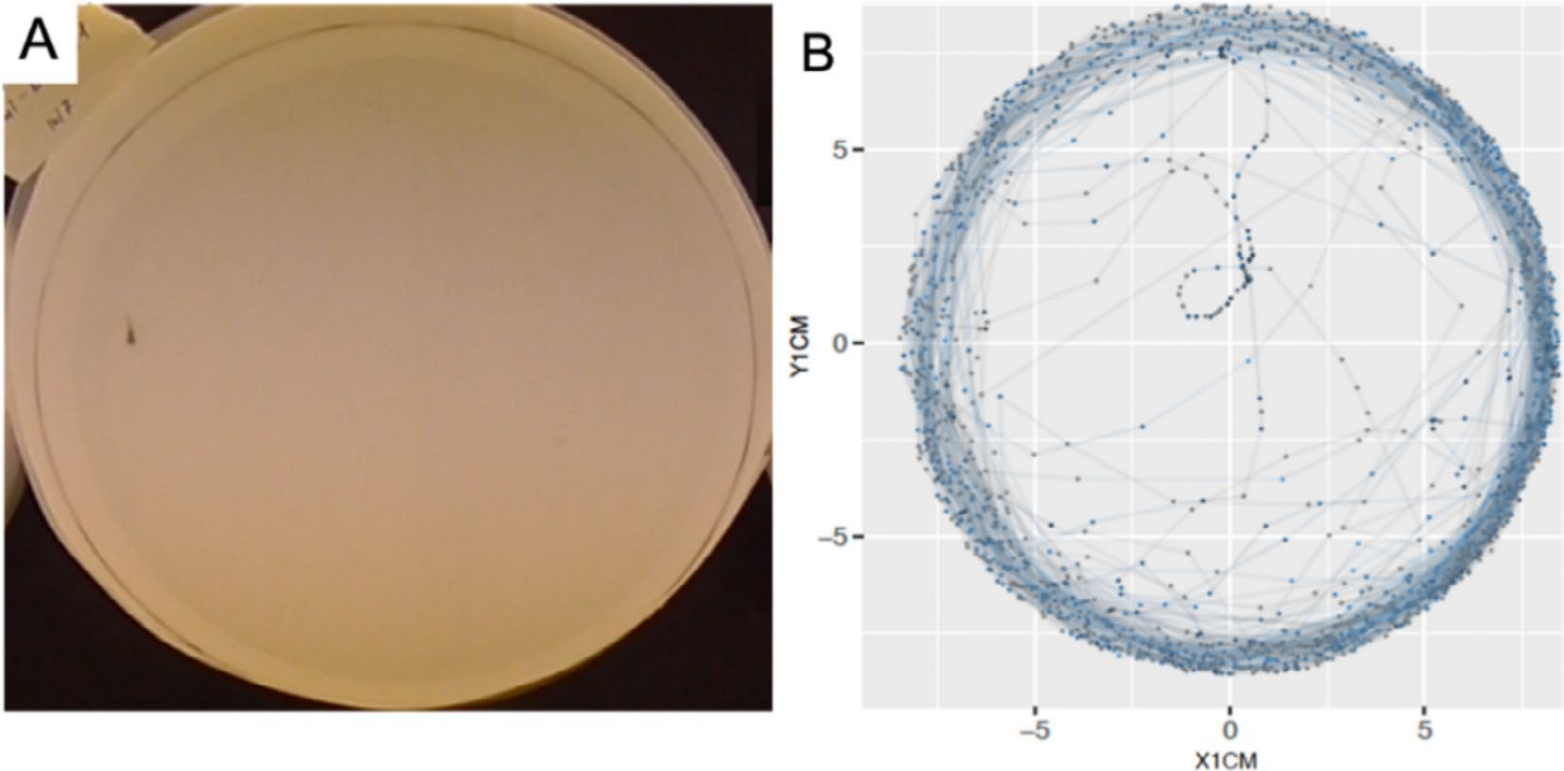
Open field test. (A) View of the arena from above and (B) example output of a tracking, showing the swimming path of a juvenile fish.

Before midday, each fish's short‐ and long‐term anti‐predator response was measured using an open field test, a common set‐up used for testing guppy behaviour (Cattelan et al. 2020; Savaşçı et al. 2021). At the start of a trial, one individual was transferred to the open field arena—a circular white arena (diameter 18 cm) containing 300 mL clean water at control temperature (26°C; Figure 1A)—and left to acclimatise for 5 min. Following acclimation, the fish was left to explore the arena for 10 min (from here on referred to as the ‘undisturbed’ phase). After these 10 min, we elicited the anti‐predator response using two stimuli: a visual stimulus followed, 1 min later, by an olfactory stimulus. The trial then continued for another 10 min (from here on these last 10 min are referred to as the ‘predator’ phase). The visual stimulus was given by switching off the light for 2 s to simulate the shadow of an overflying avian predator (Evans et al. 2007; Beppi et al. 2021). The olfactory stimulus consisted of a conspecific alarm cue that indicates injured conspecifics after a successful predator attack. This alarm cue is prepared from crushed conspecific skin following established protocols (Evans et al. 2007; Cattelan et al. 2020; Lucon‐Xiccato et al. 2020).

Fish were video‐recorded from above the arena at 25 frames per second from the start to the end of the trial. From each video, the fish's position within the arena was tracked using the software idTracker, and its coordinates were extracted for each frame (Figure 1B). The dataset was refined by calibrating and aligning the coordinates to the size and centre of the arena, by excluding tracking errors—e.g., when coordinates were positioned outside of the arena or when swimming speed > 120 cm/s—and by smoothing the swimming path. Finally, the following variables were calculated for each individual: (i) total *distance moved* in cm, (ii) *freezing time—*the proportion of time spent freezing (when velocity was less than 1 cm/s) and (iii) *swimming speed*—mean velocity in cm/s during active swimming (i.e., when moving). Each variable was calculated both as an average for each phase (i.e., undisturbed or predator phase) and also at 10 s intervals to test for temporal patterns within each phase. Each variable was also calculated separately during the 1 min following the visual predator cue.

#### 2.4.2 Capture Test and Flow Chamber Test

Following the open field test, each fish's swimming performance, in terms of its physical capacity to manoeuvre away from an approaching predator and its swimming endurance, was estimated in a capture test and a flow chamber test as follows (during the afternoon).

Predator evasion capacity was estimated using a capture test, following established protocols (Evans and Magurran 2000; Evans et al. 2004, 2007). Briefly, in this test the fish was introduced into a circular arena (49 cm diameter) containing clean water with a depth of 11 cm and left to acclimatise for 2 min. Then, the time taken (s) for an observer to catch the fish was recorded. The capture involved chasing the fish with a hand net at a consistent speed. The test began when the fish was positioned centrally in the arena, and the time required for its capture was recorded using a stopwatch. This ‘capture time’ has been widely used in this species to assess the escape abilities of fish, both in juveniles and adults (Evans and Magurran 2000; Evans et al. 2004, 2007; Gasparini et al. 2013).

Swimming endurance, a proxy for health condition, was estimated by measuring the critical swimming speed of each fish in a flow chamber, using a previously established protocol (Nicoletto 1991; Breedveld et al. 2023). Each fish was placed into a swimming chamber (a transparent PVC pipe measuring 50 cm in length and 1.5 cm in diameter), through which water was pumped at a steady linear flow using an aquarium pump. The water velocity was initially set at 7 cm s^−1^ and increased by 3 cm s^−1^ every minute, until the fish could no longer maintain its position against the current and was swept out of the chamber into a water tank below. The total swimming time, the highest water velocity and the time spent swimming at the highest velocity were recorded for each fish. These measurements were used to calculate its critical swimming speed after Brett (1964). Critical swimming speed is defined as the maximum speed a fish can sustain for a set period (Brett 1964), and is a measure of endurance (Gordon et al. 2015).

### 2.5 Statistical Analysis

The effects of a heatwave on anti‐predator responses and swimming performance of juvenile *P. reticulata* (*N* = 103; 52 heatwave, 51 control) were investigated with linear and generalized linear mixed‐effects models (LMMs and GLMMs) using the package lme4 (Bates et al. 2015) in R version 1.4.0 (R Core Team 2021). All statistical models included treatment (heatwave or control) as a fixed factor and family ID as a random factor to account for the non‐independence of data coming from siblings. Treatment tank was also included as a random factor but removed from the final models, as it was not significant in any analyses. Body condition (Fulton's index) was tested as a covariate in all models, but was not included, as it was not significant in any analysis, nor affected by treatment (*F* = 0.16_(1,83.1)_, *p* = 0.688). GLMMs were tested for overdispersion using the function dispersion_glmer from the package blmeco (Korner‐Nievergelt et al. 2015) and corrected where necessary. The significance of fixed effects in LMMs was calculated from F statistics with the lmerTest package (Kuznetsova et al. 2017) and Satterthwaite's approximation to calculate the denominator degrees of freedom. The significance of fixed effects in GLMMs was calculated from chi‐squared statistics, using Wald chi‐squared tests from the car package (Fox and Weisberg 2019). Mean ± SE are reported unless otherwise indicated.

To analyse the short‐term anti‐predator response (following the visual stimulus), we used each individual's value for the three activity variables (total distance moved, freezing time, mean swimming speed) for 1 min following the stimulus. Total distance moved and mean swimming speed were both square‐root transformed to improve the normality of the residuals and analysed using LMMs. Freezing time was analysed as a proportion using a GLMM fitted with a binomial family distribution and a two‐column matrix (using c‐bind) of the number of seconds freezing and the number of seconds moving as a response variable.

To analyse the long‐term anti‐predator response (following the olfactory stimulus), we took a two‐step approach: overall reaction (i) and temporal response (ii). (i) The overall reaction to this stimulus was analysed by comparing activity during the 10‐min predator phase to activity during the undisturbed phase. LMMs were used for total distance moved and swimming speed (square‐root and log‐transformed respectively, to improve normality of the residuals), and a GLMM with binomial family distribution was used for freezing time (proportion, as described above). Models included treatment (heatwave or control) and phase (undisturbed or predator) as fixed factors, and individual ID as random to account for repeated measures of the same individual. (ii) The second step was to determine the temporal changes in behaviours after the exposure to the stimulus during the predator phase, by determining individual reactions at 10‐s intervals (for additional analyses of the temporal response, i.e., acclimation time, during the undisturbed phase see Supplement S1). LMMs were used for total distance moved and swimming speed (square‐root and log‐transformed, respectively, to improve normality of the residuals), and a GLMM with binomial family distribution was used for freezing time (proportion, as described above). Models included treatment and time (per 10 s) as fixed effects, and individual ID as random to account for repeated measures of the same individual. Body size was tested as a covariate in these models, but not included due to non‐significance.

Capture time (log‐transformed to improve normality of the residuals) and swimming endurance were analyzed using LMMs, with treatment (heatwave or control) as a fixed factor. Body size was included as a covariate in these models.

## 3 Results

### 3.1 Anti‐Predator Response

#### 3.1.1 Short Term Anti‐Predator Response

**FIGURE 2:**
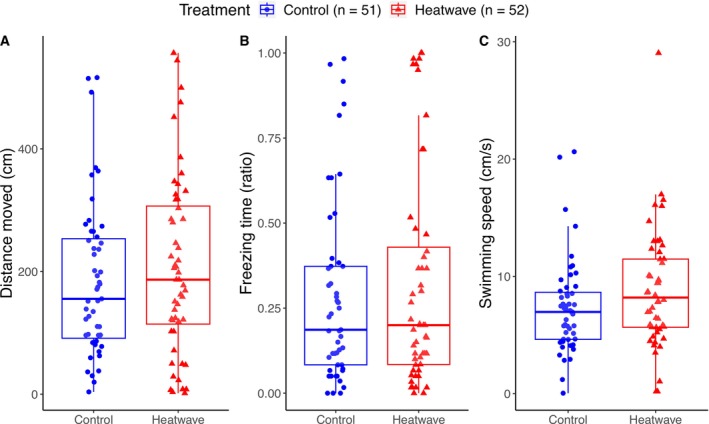
Anti‐predator response to a visual predator cue. Boxplots showing the anti‐predator response of juvenile guppies, *Poecilia reticulata*, during 1 min following the visual stimulus, measured by three different variables: Total distance moved (A), freezing rate (B), and swimming speed (C). Controls are represented as blue circles and treated individuals (heatwave) as red triangles. Boxes represent the interquartile range (IQR), with whiskers extending to 1.5 times the IQR from the first and third quartiles.

Treatment (heatwave or control) did not affect the juveniles’ response to the visual cue, in terms of their total distance moved (*F* = 0.21_(1,82.8)_, *p* = 0.648), freezing time (*χ* ^2^ = 0.50_(1)_, *p* = 0.480) or swimming speed (*F* = 2.22_(1,78.5)_, *p* = 0.141), during the minute following the stimulus (Figure 2).

#### 3.1.2 Long‐Term Anti‐Predator Response

**FIGURE 3:**
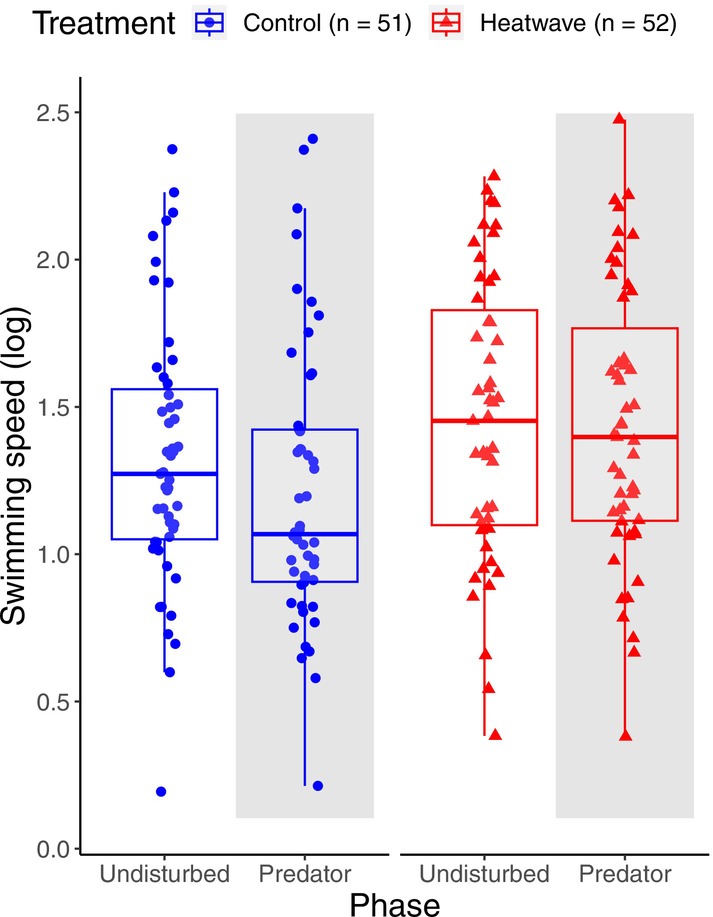
Overall swimming speed response to the chemical stimulus. Boxplot showing the average swimming speed of juvenile guppies, *Poecilia reticulata*, from a control (blue circles) or a heatwave (red triangles) group, during the 10 min undisturbed phase (clean water; background in plot not shaded) and the 10 min predator phase (conspecific alarm cue present in the water; plot background shaded grey). Boxes represent the interquartile range (IQR), with whiskers extending to 1.5 times the IQR from the first and third quartiles.

Overall activity comparisons between the predator and undisturbed phases (see Table S1 for descriptive statistics) confirmed that the chemical stimulus induced a response in the fish in all the variables considered (all *p* < 0.003, Table S2). The total distance moved was lower (phase: *p* < 0.001) and the freezing time higher (phase: *p* < 0.001) in the predator phase than in the undisturbed phase, but there was no effect of treatment (heatwave or control) or the interaction between treatment and phase (Table S2, Figures S1A and S1B). Similarly, average swimming speed was lower in the predator phase than in the undisturbed phase (phase: *p* = 0.003), but there was also a significant effect of the treatment and the interaction between treatment and phase (treatment: *p* = 0.032, interaction: *p* = 0.034, see Table S2, Figure 3). This indicates that the heatwave group exhibited (i) an overall higher swimming speed (both before and after the predator cue) compared to the control group, and (ii) an attenuated reaction to the stimulus, characterised by a smaller reduction in speed after the predator cue.

**FIGURE 4:**
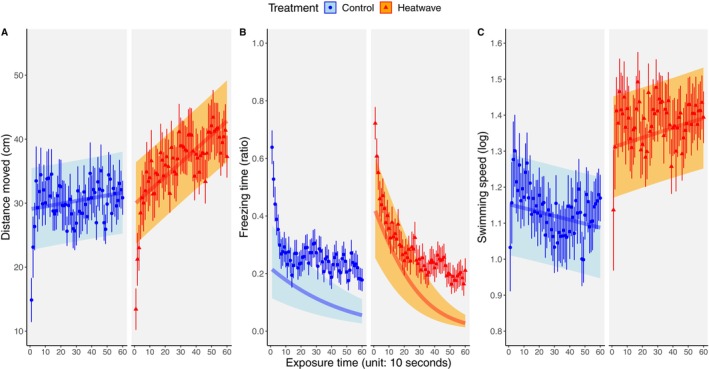
Anti‐predator response to the olfactory alarm cue. The temporal response, in terms of distance moved (A), freezing rate (B), and swimming speed (C), of juvenile guppies, *Poecilia reticulata*, from a control (blue circles, *n* = 51) or a heatwave (red triangles, *n* = 52) treatment, to a conspecific olfactory alarm cue. Each variable is plotted as mean ± SE per 10 s of exposure during the 10‐min predator phase. The model's predicted slope (smoothed estimates based on the fitted model; thick transparent line) and confidence intervals (SE of the predicted values; shade around the slope) are also shown. (see Figure S2 for responses to exposure time in the undisturbed phase.)

The temporal analyses within the predator phase revealed a significant effect of treatment on the fish’ long‐term anti‐predator response. Distance moved was significantly affected by time and the interaction between treatment and time (time: *p* < 0.001, interaction: *p* < 0.001, Table 1, Figure 4A). Specifically, there was an increase in the distance moved with time, which occurred faster (i.e., there was a steeper slope) in the heatwave than in the control treatment. A similar temporal pattern was found for freezing time. This was significantly affected by time and the interaction between treatment and time (time: *p* < 0.001, interaction: *p* < 0.001, Table 1, Figure 4B), showing that freezing time decreased with the time elapsed from the moment in which the predator cue was introduced into the arena (i.e., the fish resume normal activity with time), but this occurred sooner (steeper slope) in the heatwave group compared to the control. Average swimming speed was significantly affected by the interaction between treatment and time and was marginally significant for treatment (interaction: *p* < 0.001, treatment: *p* = 0.053, Table 1, Figure 4C). Inspection of the two slopes (Figure 4C) suggests that swimming speed increased with time in the heatwave group while it decreased in the control.

**TABLE 1:** Temporal response in the predator phase: The effect of heatwave treatment on distance moved, freezing time, and swimming speed in juvenile guppies, *Poecilia reticulata* .

	Temporal anti‐predator response (predator phase)								
	Treatment			Time			Treatment × Time		
	Estimate ± SE	*F*(denDF)/*χ* ^2^	*p*	Estimate ±se	*F*(denDF)/*χ* ^2^	*p*	Estimate ±se	*F*(denDF)/*χ* ^2^	*p*
Distance moved	−0.179 ± 0.35	0.26 (92.8)	0.614	0.010 ± 0.002	265.67 (6070)	**< 0.001**	0.017 ± 0.002	56.02 (6070)	**< 0.001**
Freezing time	0.994 ± 0.49	*0.09*	0.764	−0.026 ± 0.003	*408.04*	**< 0.001**	−0.029 ± 0.004	*53.58*	**< 0.001**
Swimming speed	0.158 ± 0.08	3.84 (89.0)	0.053	−0.001 ± 0.000	0.15 (5320.5)	0.700	0.002 ± 0.001	20.50 (5320.8)	**< 0.001**

*Note:* The anti‐predator response by juvenile *P. reticulata* (*N* = 103), subjected to a heatwave or control treatment, in terms of three variables (distance moved, freezing time and swimming speed) analysed as temporal changes in fish behaviour in an open field during 10 min following the release of a conspecific alarm cue (i.e., during the predator phase). The table presents the results from models testing each variable in response to the temperature TREATMENT (control or heatwave), the TIME in the predator phase (in 10 s units), and their interaction. *F* statistics for LMM's are given with their corresponding denominator degrees of freedom (denDF) and *χ* ^2^ statistics for GLMM's are italicised. Significant effects are in bold.

### 3.2 Capture Test and Flow Chamber Test

Capture time, i.e., predator evasion capacity, and swimming endurance were not significantly affected by treatment (capture time: *F* = 1.42_(1,84.7)_, *p* = 0.236; swimming endurance: *F* = 2.60_(1100)_, *p* = 0.110). Swimming endurance was positively correlated with body size (estimate = 0.915, *F* = 15.051_(1100)_, *p* < 0.001) and capture time tended to increase with body size (estimate = 0.196, *F* = 3.53_(1,71.4)_, *p* = 0.064).

## 4 Discussion

Our results show that a 5‐day heatwave can alter anti‐predator responses in juvenile fish. While we found no effect on the fish's condition (measured as swimming performance in a flow chamber and a capture test), their responses to predator cues were altered. Specifically, the heatwave did not affect the short‐term reaction to the visual stimulus, but did impact the longer‐term reaction to the conspecific alarm cue, by decreasing the fish's freezing and movement response. After being exposed to the olfactory predator cue, juveniles that experienced a heatwave thereby exhibited a quicker recovery to pre‐cue (baseline) behaviour compared to the controls. By inducing inappropriate anti‐predator responses, heatwaves could increase the predation risk of prey by, for example, increasing detection rates (Cordonnier et al. 2023), and thus alter prey survival and predator–prey interactions, with potential cascading effects on communities of interacting organisms (Pintanel et al. 2021).

Both heatwave and control fish responded to the predator threat, as evidenced by their freezing behaviour in response to the visual cue, and changes in all three activity variables in response to the olfactory cue. This confirms that the experimental cues were effective in eliciting a behavioural anti‐predator response, in agreement with long‐standing research on anti‐predator behaviour in guppies (e.g., Seghers 1974; Kelley and Magurran 2003a). The overall responses to the predator cues followed the expected pattern: in the presence of a cue, individuals spent more time exhibiting freezing behaviour and generally moved less and at a lower speed than in their absence. These responses are in line with typical anti‐predator behaviours of fishes (Kelley and Magurran 2003b). The overall reduction in swimming speed was treatment dependent, as shown by the significant interaction between treatment and phase. This indicates that heatwaves changed the fish's anti‐predator response to the olfactory alarm cue; specifically, it weakened their reaction of slowing down. As the interaction term is significant, the interpretation of the main effects must be approached with caution. Therefore, our discussion will mainly focus on the interactions rather than the single effects.

In the temporal responses to the olfactory predator cue, as shown in Table 1, all three variables showed a significant interaction between treatment and time, indicating a different temporal pattern in the response to a predator threat between heatwave‐exposed and control fish. Specifically, heatwave fish showed a weaker longer‐term response to the alarm cue, as shown by the interactions (Figure 4). Although fish from both groups initially responded to the alarm cue in a similar way, heatwave fish exhibit a shorter latency to resume normal activity, recovering more quickly than controls to their pre‐cue behaviour. The quicker resumption of activity by heatwave‐exposed fish under predation risk could increase the chance of detection by predators, as more active prey are usually more conspicuous to predators (Lima and Dill 1990; Krause and Godin 1995). Thereby, a too rapid return to normal activity could be detrimental for survival, especially when considering that temperature can also influence other anti‐predator behaviours in the guppy, such as shoaling and the distance kept from a predator (Weetman et al. 1999; Zanghi et al. 2023).

In contrast to the results on the response to the olfactory cue, treatment did not affect the fish's short‐term behavioural reaction to the visual cue. This suggests that heatwaves have a larger impact on guppies' responses to olfactory cues than to visual ones, in line with evidence that environmental stressors often have sensory modality‐specific effects, potentially affecting one sensory modality while leaving others relatively unaffected (Rivest et al. 2019). Another possibility is that prey organisms exposed to heatwaves may prioritise certain sensory pathways (e.g., visual) over others (e.g., olfactory), as a strategy to allocate their limited energy towards cues that provide more reliable information about predation risk. For guppies and other prey species in aquatic environments, visual and chemical cues provide different types of information about the local predation risk (Kelley and Magurran 2003b). Visual cues are spatially and temporally reliable—they indicate that the predator must be close—and are therefore considered more immediate and riskier. In contrast, chemical cues linger in the environment and can be distorted by currents (Brown 2003; Stephenson 2016). Moreover, visual cues often induce a stronger innate response (Kelley and Magurran 2003a). Reactions to chemical cues are concentration‐dependent, i.e., higher concentrations indicate a greater threat (Brown 2003), and show ontogenetic variation (Xia et al. 2017). Thus, under heat stress, some prey fish may prioritise their attention to more risky visual cues at the cost of reduced attention to olfactory predator cues, potentially explaining our findings that heatwaves reduced the fish's responses to the conspecific alarm cue but not to the visual cue. Another possible explanation for the difference in the observed effects between visual and olfactory cues is the difference in sampling duration. While visual cues typically elicit short‐term responses, a longer sampling period might have captured additional behavioural changes. Future studies could explore this possibility by extending the observation period for visual stimuli.

While our main aim was to determine whether or not heatwaves have an effect on anti‐predator responses, rather than identify the underlying mechanisms, we may speculate about potential mechanisms for the observed effects. The attenuated anti‐predator responses in heatwave‐exposed juvenile guppies could result from heat‐stress induced changes in their physical condition (Dittmar et al. 2014; Ritchie and Friesen 2022), be a side effect of increased metabolic rate (Biro et al. 2010; Culumber 2020), or derive from changes in their perception or interpretation of predator cues (Rivest et al. 2019). Since we found no differences between control and heatwave fish in swimming performance or body condition, it is unlikely that the observed differences in behaviour resulted from a reduced physical condition in the heatwave group. Another possibility is that the heatwave increased the metabolic rate of fish, leading to a shift towards more risk‐taking behaviour. This behavioural change could explain the shorter latency to resume activity after a predator threat under our experimental setup. In support of this explanation, a study on *Pseudoxiphophorus jonesii*, a small freshwater fish from the same family as *P. reticulata* , found that individuals exposed to elevated temperatures for 3 days showed increased activity and a greater tendency to engage in risky behaviour (Culumber 2020). However, other studies show that organisms can acclimate to warmer temperatures by reducing their metabolic rate (e.g., Stahlschmidt and Glass 2020), which suggests that this explanation may not apply in all cases. These possibilities highlight the need for further research to better understand the links between metabolic changes, risk‐taking, and predator avoidance behaviour. A third possible explanation is that the quicker resumption of activity after the predator threat in the heatwave group results from some heat‐stress induced impairment in the sensory pathways or in cognitive processes, i.e., the mental processes involved in perceiving and processing cues (Rivest et al. 2019; Soravia et al. 2021). There are two potentially affected cognitive mechanisms: attentional, i.e., those involved in perceiving and processing information and decision‐making, i.e., those used to determine the appropriate response (Moiroux et al. 2015; Soravia et al. 2021). Heatwave‐exposed fish initially reacted to the predator cue as the control fish, indicating they could perceive it, but thereafter showed a reduced response. This suggests that while heatwaves may not impair attentional mechanisms, they could affect decision‐making processes. Decision‐making depends on the neural transmission (as electrochemical signal) of cue information from sensory organs to sensory areas of the brain, where it is processed to generate a response, such as an anti‐predator response (Rivest et al. 2019). Heat stress can compromise neurotransmission directly, thereby affecting decision‐making (Rivest et al. 2019), or indirectly by influencing neuronal development during the sensitive stage of ontogeny, which can alter the number and function of synapses in the brain (Santana et al. 2020). Since our fish were subjected to a heatwave during early life, heat stress may indeed have affected the development of their decision‐making mechanisms. Similarly, other types of stressors have been shown to modify cognitive development (e.g., nutritional stress; Farrell et al. 2016). Finally, physiological stress induced by heatwaves could have led to higher risk taking (Gutiérrez et al. 2023), or defence systems activated by heat stress could have diverted energy or attention away from predator cues, thereby changing anti‐predator responses (Godin and Sproul 1988). Future studies with a physiological approach should help to better distinguish between alternative mechanisms underlying our observed differences in anti‐predator responses after heatwaves.

Our findings align with a small but growing body of research indicating that heatwaves can alter the behavioural responsiveness of prey to predators (Allan et al. 2015; Cordonnier et al. 2023; Gutiérrez et al. 2023; Jawad et al. 2024). Such changes may reduce prey escape efficiency, increasing their susceptibility to predation. Clearly, the extent of this effect depends also on the heat sensitivity of their predators. When predators and prey have similar heat tolerances, heatwave‐induced changes may be symmetrical, resulting in minimal effects on biotic interactions. More often, though, temperature affects the physiology and behaviour of predators and their prey differently (Anderson et al. 2001; Barton 2011; Smolinský and Gvoždík 2014; Allan et al. 2015; Pintanel et al. 2021; Tscholl et al. 2022), making it likely that heatwaves will influence predator–prey dynamics. Additionally, long‐term responses of predators to a warmer environment (e.g., acclimation) could modify the top‐down effects that predators have on prey communities (Grigaltchik et al. 2012). This highlights the importance of assessing both the short‐ and long‐term effects of heatwaves on species and trophic interactions, to better understand the stability, resilience, and resistance of ecological communities in response to climate change.

## 5 Conclusion

Our study demonstrates that heatwaves modify the anti‐predator response of juvenile guppies, *P. reticulata* , to an olfactory predator cue. Specifically, heatwave‐exposed juveniles exhibited a weakened reaction to a conspecific alarm cue, evidenced by their faster recovery to pre‐cue activity levels compared to control fish. These findings reveal that heatwaves can significantly influence prey behaviour, potentially increasing their vulnerability to predators and elevating predation risk. By altering prey responsiveness to predator cues, heatwaves—which are becoming increasingly frequent and severe—may ultimately impact prey survival in the wild, with broader implications for predator–prey dynamics and communities of interacting organisms. Our results not only illustrate that heatwaves can influence critical behaviours with potential ecological impacts, but also underscore the need for future research into the long‐term persistence of such behavioural alterations. Understanding the duration and plasticity of behavioural changes will be essential for assessing their full impact on population dynamics and ecosystem functioning in a warming climate.

## Author Contributions

**Merel C. Breedveld:** conceptualization (equal), data curation (equal), formal analysis (equal), funding acquisition (equal), investigation (equal), methodology (equal), project administration (equal), resources (equal), software (equal), supervision (equal), validation (equal), visualization (equal), writing – original draft (equal), writing – review and editing (equal). **Oliviero Borgheresi:** investigation (equal), writing – review and editing (equal). **Alessandro Devigili:** investigation (equal), writing – review and editing (equal). **Clelia Gasparini:** conceptualization (equal), supervision (equal), writing – review and editing (equal).

## Ethics Statement

All experiments were carried out with the approval of the University of Padova's Animal Ethics Committee and the national authorities (approval no: 313/2022‐PR).

## Conflicts of Interest

The authors declare no conflicts of interest.

## Supporting information


Data S1.

## Data Availability

Data is available on dryad: https://doi.org/10.5061/dryad.gb5mkkx13.
