# Supplementary material for: Stunned by a Heatwave: Experimental Heatwaves Alter Juvenile Responsiveness to the Threat of Predation
Source: Ecol Evol. 2025 May 14;15(5):e71447. doi: 10.1002/ece3.71447 (PMC12077929; doi:10.1002/ece3.71447)
Supplement: Supplementary file 1 — Data S1. [file ECE3-15-e71447-s001.pdf]

**Supporting information for:**

# Stunned by a heatwave: experimental heatwaves alter juvenile responsiveness to the threat of predation

M.C. Breedveld, O. Borgheresi, A. Devigili, C. Gasparini

**Table S1 Mean activity values.** Behaviour in an open field of juvenile guppies, *P. reticulata* (N = 103), priorly subjected to a heatwave or control, in terms of three movement variables (distance moved, freezing time, swimming speed). Mean values ± standard errors (SE) are given for each TREATMENT group (control or heatwave) in each 10-minute PHASE (undisturbed or predator, that is, prior to and after the release of a predator cue).

| | **CONTROL (N = 51)** | | **HEATWAVE (N = 52)** | |
|---|---|---|---|---|
| | UNDISTURBED PHASE | PREDATOR PHASE | UNDISTURBED PHASE | PREDATOR PHASE |
| Distance moved (cm) | 2350 ± 159 | 1807 ± 147 | 2570 ± 184 | 2163 ±199 |
| Freezing time (ratio) | 0.11 ± 0.02 | 0.26 ± 0.03 | 0.13 ± 0.03 | 0.28 ± 0.03 |
| Swimming speed (log, cm/s) | 1.33 ± 0.06 | 1.21 ± 0.07 | 1.46 ± 0.07 | 1.44 ± 0.06 |

**Table S2. Overall response to the stimulus.** Models testing the overall anti-predator response by juvenile guppies, *P. reticulata* (N = 103), subjected to a heatwave or control, in terms of three movement variables (distance moved, freezing time, swimming speed), by comparing their behaviour in an open field prior to and after the release of a conspecific alarm cue (i.e. 10-minute undisturbed phase versus 10-minute predator phase). The table presents the results from models testing each variable in response to the temperature TREATMENT (control or heatwave), the PHASE (undisturbed or predator), and their interaction. F statistics for LMM's are given with their corresponding denominator degrees of freedom (denDF) and χ2 statistics for GLMM's are underlined. Significant effects and trends are printed in bold.

| | **Mean anti-predator response** | | | | | | | | |
|---|---|---|---|---|---|---|---|---|---|
| | **TREATMENT** | | | **PHASE** | | | **TREATMENT x PHASE** | | |
| | estimate ±se | *F*(denDF)/$\chi^2$ | *p* | estimate ±se | *F*(denDF)/$\chi^2$ | *p* | estimate ±se | *F*(denDf)/$\chi^2$ | *p* |
| Distance moved | 1.720 ±2.54 | 1.09 (84.6) | 0.300 | -6.517 ±1.17 | 48.17 (101.0) | **<0.001** | 1.560 ±1.65 | 0.89 (101.0) | 0.347 |
| Freezing time | 0.138 ±0.32 | <u>0.24</u> | 0.623 | 1.439 ±0.19 | <u>123.03</u> | **<0.001** | 0.010 ±0.26 | <u>0.00</u> | 0.969 |
| Swimming speed | 0.130 ±0.09 | 4.78 (82.8) | **0.032** | -0.122 ±0.03 | 9.37 (99.3) | **0.003** | 0.099 ±0.05 | 4.60 (99.3) | **0.034** |

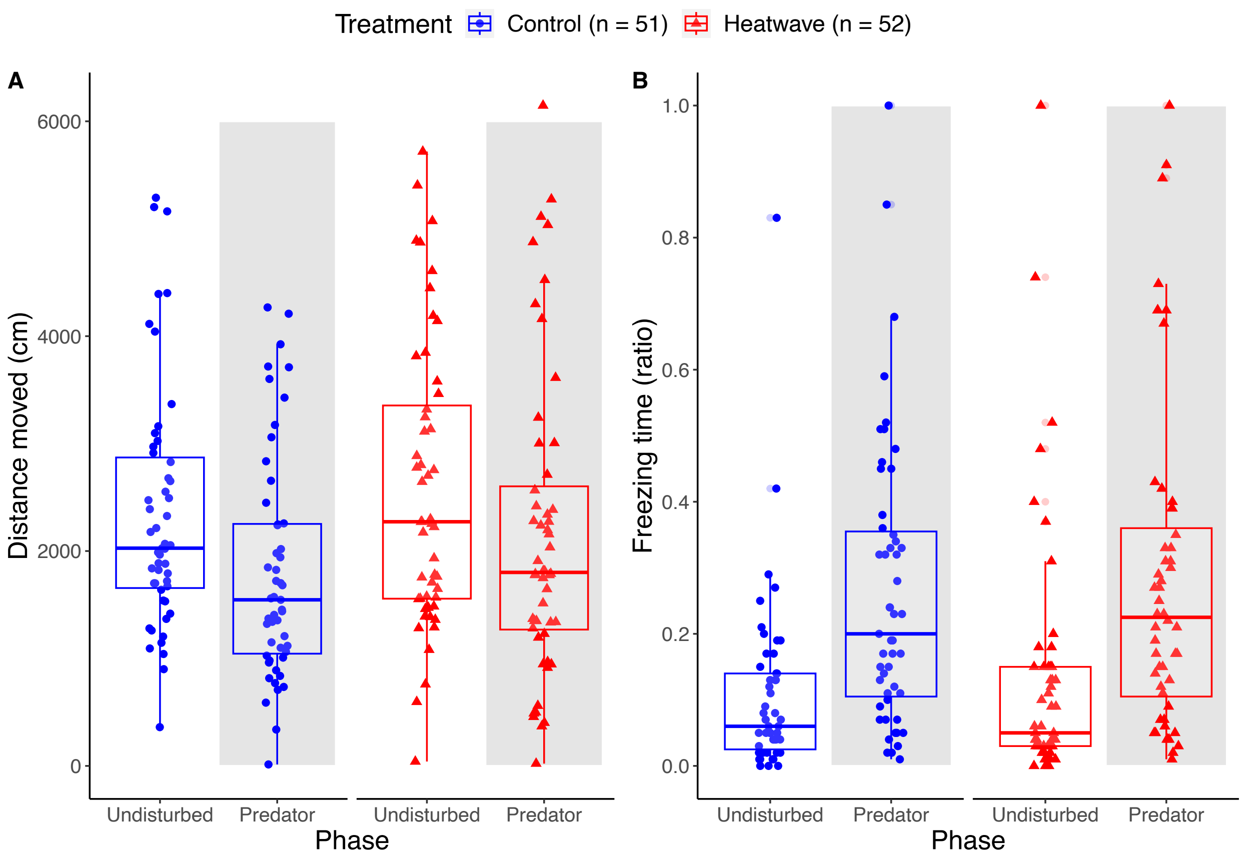


**Figure S1. Overall response to the chemical stimulus.** Boxplot showing the total distance moved (A) and freezing rate (B) of juvenile guppies, *P. reticulata,* from a control (blue circles) or a heatwave (red triangles) treatment, during a 10-minute undisturbed phase (clean water; background in plot not shaded) and a 10-minute predator phase (conspecific alarm cue present in the water; plot background shaded grey). Boxes represent the interquartile range (IQR), with whiskers extending to 1.5 times the IQR from the first and third quartiles.

## S.1 Temporal response in the UNDISTURBED phase

The temporal analyses of *distance moved* within the undisturbed phase (which was, in total, higher than in the predator phase; see section 3.1.2) showed an interaction (weaker than in the predator phase) between treatment and time (Table S1, Fig. S1 top). The interaction points to a slight increase with time in moving distance in the heatwave group, in contrast to no temporal change in moving distance in the control group.

*Freezing time* within the undisturbed phase (which was, in total, lower than in the predator phase; see section 3.1.2) was affected by time and the interaction between treatment and time (Table S1, Fig. S1 centre), pointing to a very slight increase with time in freezing rate in the control group but not in the heatwave group.

*Swimming speed* within the undisturbed phase (which was, in total, higher than in the predator phase for the control group only; see section 3.1.2) was affected by time and the interaction between treatment and time (Table S1, Fig. S1 bottom), pointing to an increase with acclimation time in swimming speed in the heatwave group, but not in the control group.

**Table S3. Temporal response in the undisturbed phase.** The temporal acclimation response by juvenile guppies, *P. reticulata* (N = 103) – recently subjected to a heatwave or control treatment – in terms of three movement variables (distance moved, freezing time, swimming speed), by analysing temporal changes in behaviour in an open field for 10 minutes following initial acclimation (i.e. during the undisturbed phase). The table presents the results from (G)LMM models testing each variable in response to the temperature TREATMENT (control or heatwave), the TIME in the undisturbed phase (in 10 second units), and their interaction. F statistics for LMM's are given with their corresponding denominator degrees of freedom (denDF) and $\chi 2$ statistics for GLMM's are underlined. Significant effects and trends are printed in bold.

| | **Temporal response (undisturbed phase)** | | | | | | | | |
|---|---|---|---|---|---|---|---|---|---|
| | **TREATMENT** | | | **TIME** | | | **TREATMENT x TIME** | | |
| | estimate ±se | $F$(denDF)/$\chi^2$ | $p$ | estimate ±se | $F$(denDF)/$\chi^2$ | $p$ | estimate ±se | $F$(denDF)/$\chi^2$ | $p$ |
| Distance moved | 0.053 ±0.33 | 0.03 (88.5) | 0.871 | -0.001 ±0.001 | 0.99 (6074) | 0.320 | 0.004 ±0.002 | 4.64 (6074) | **0.031** |
| Freezing time | 0.432 ±0.39 | 0.16 | 0.693 | 0.009 ±0.002 | 8.61 | **0.003** | -0.009 ±0.003 | 8.86 | **0.003** |
| Swimming speed | 0.064 ±0.09 | 0.53 (84.7) | 0.470 | -0.000 ±0.000 | 3.70 (5882.2) | **0.054** | 0.002 ±0.000 | 15.76 | **<0.001** |

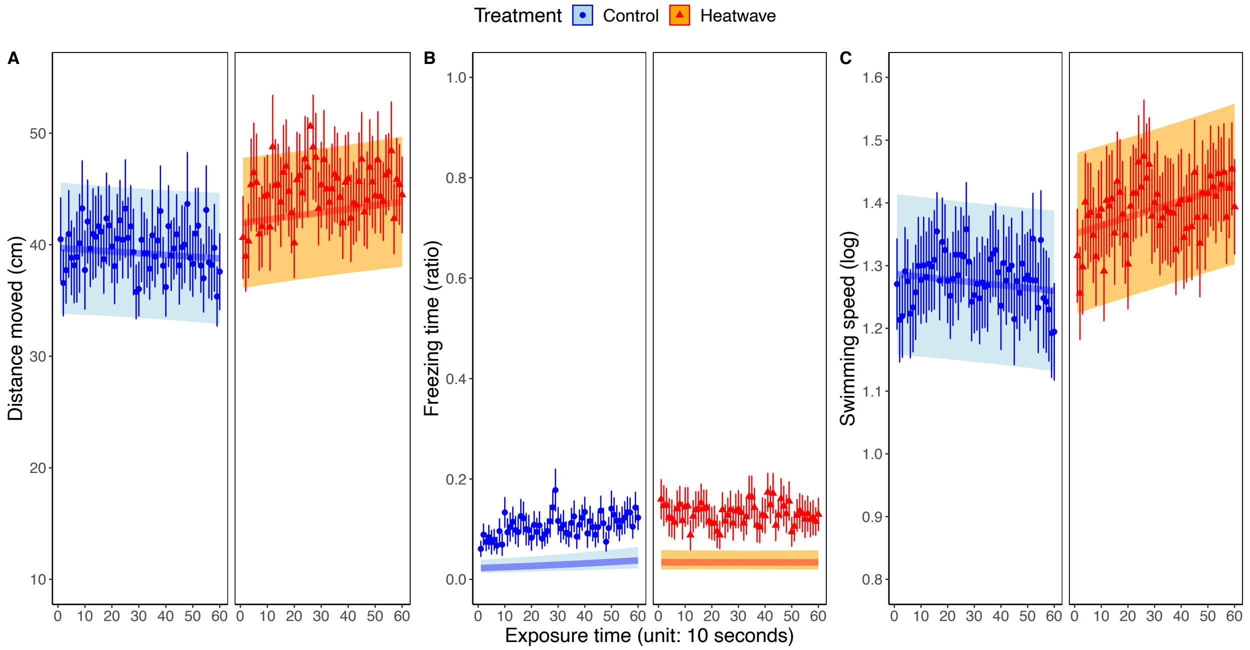


**Figure S2. Temporal response to acclimation in an open field (before any predator-related stimulus).** Distance moved (A), freezing rate (B), and swimming speed (C), of juvenile guppies, *P. reticulata*, from a control (blue circles, *n* = 51) or a heatwave (red triangles, *n* = 52) treatment. Each variable is plotted as mean ± SE per 10 seconds of exposure time during the 10-minute undisturbed phase. The model's predicted slope (smoothed estimates based on the fitted model; thick transparent line) and confidence intervals (SE of the predicted values; shade around the slope) are also shown.
